# Defining the Efficiency of Manual Ventilation: A Comprehensive Systematic Review

**DOI:** 10.1155/emmi/9961736

**Published:** 2025-02-18

**Authors:** Julian Lasik, Tomasz Kłosiewicz, Mateusz Puślecki

**Affiliations:** Department of Medical Rescue, Chair of Emergency Medicine, Poznan University of Medical Sciences, Poznan, Poland

**Keywords:** bag-valve-mask, BVM, efficiency, manual ventilation, performance

## Abstract

Manual ventilation is an essential skill for healthcare professionals, especially in emergency and resuscitation situations where mechanical ventilation may not be immediately available. However, improper manual ventilation can lead to serious complications such as barotrauma (lung injury caused by excessive pressure), hypoventilation (leading to insufficient oxygenation), hyperventilation (which can cause respiratory alkalosis and reduced cerebral blood flow), and gastric insufflation (which increases the risk of aspiration). This review aimed to analyze the definitions and methods used to assess manual ventilation efficiency in recent studies. A systematic database search was conducted for the period between 2014 and 2023. The primary inclusion criterion was the assessment of manual ventilation quality in adults. Out of 47 identified studies, eight met the inclusion criteria in the review. Most of the reviewed studies focused on key ventilation parameters including tidal volume and ventilation rate, which are critical for ensuring adequate ventilation. However, we found considerable variability in how “effective ventilation” was defined. This review highlights the approach that considers both extrinsic and intrinsic factors as a potentially more comprehensive method for assessing manual ventilation quality. This approach may offer a more consistent and effective framework for ensuring safe and efficient manual ventilation practices.

## 1. Introduction

Manual ventilation has been extensively studied in recent years, underscoring its importance in medical care. However, the proficiency of healthcare personnel in hospital and prehospital settings in performing effective ventilation varies significantly, potentially leading to significant challenges [1, 2]. Ventilation with a self-expanding bag-valve-mask (BVM) and an oxygen reservoir is the primary method performed by less-trained personnel as well as experienced healthcare specialists in hospital and prehospital settings.

Inadequate ventilation, characterized by delivering too few breaths with insufficient volume, can lead to hypoxemia, hypercapnia, and progressive acidosis [3–5]. Conversely, high tidal volumes or high pressure, delivered may cause lungs injury, pneumothorax, mediastinal emphysema, and subcutaneous emphysema [6]. Other complications include gastric insufflation and risk of aspiration [7–9]. These potential complications highlight the critical need to identify precise determinants of effective ventilation.

Most recent studies on manual ventilation have been conducted under controlled laboratory conditions, often using manikin simulations that lack the physiological variables of the human body. The growing interest in this area is driven by the potential complications associated with improperly performed ventilation and pressure to introduce new medical products to a market considered one of the fastest-growing industries. Most authors refer to recommendations of the International Liaison Committee on Resuscitation (ILCOR) for performing manual ventilation. However, ILCOR guidelines do not specify how to evaluate ventilation in controlled simulation settings, providing only general recommendations on safe ventilation rates and tidal volumes [10–13]. As a result, some researchers have developed own parameters for analysis to address this gap.

### 1.1. Aim of the Study

This review aimed to analyze the definitions and methods used to assess manual ventilation efficiency in recent studies.

## 2. Materials and Methods

### 2.1. Data Search

Two independent researchers, JL and TK, searched three electronic databases: Embase, Pubmed, and Cochrane from 2014 to April. For this systematic review, two concepts have been created. Concept one was efficiency and concept two was manual ventilation. The first concept consisted of the following keywords: performance, efficiency, and productivity. The second concept consisted of manual ventilation. The researchers used keywords such as manual ventilation and BVM in this concept. A MeSH strategy was also used: Ventilation: (“Respiration, Artificial/adverse effects” [Majr:NoExp] OR “Respiration, Artificial/instrumentation” [Majr:NoExp] OR “Respiration, Artificial/methods” [Majr:NoExp]). Each of these terms was used individually as well as in combination. Concept one and concept two were combined to form the final search strategy.

The inclusion criterion for this review was the analysis of the effectiveness of manual ventilation using any method of measurement. Papers written in languages other than English, focused on pediatric ventilation, and related to COVID-19 pandemic were excluded. In addition, studies that provided incomplete descriptions of methods for assessing ventilation effectiveness or insufficient data analysis were also excluded. For research purposes, data were gathered from the European Resuscitation Council (ERC) Guidelines 2015 and 2021, as well as the American Heart Association's (AHA) Guidelines 2015 and 2020.

### 2.2. Legal Issue

The study protocol was reviewed by Bioethics Committee at Poznan University of Medical Sciences. According to the Polish Law and Good Clinical Practice, our study was not qualified as a medical experiment (decision number KB-362/24).

## 3. Results

### 3.1. Eligibility Criteria

The database search using the presented strategy yielded the following results: Embase: 682 results, Cochrane Library: 958 results, and PubMed: 3985 results. A total of 538 studies matched the set parameters. After removing duplicates, 503 unique records were identified. Among the remaining 456 papers, 49 were related to pediatrics and 12 explored ventilation in the context of the COVID-19. Moreover, 395 papers were unrelated to ventilation.

**Figure 1:**
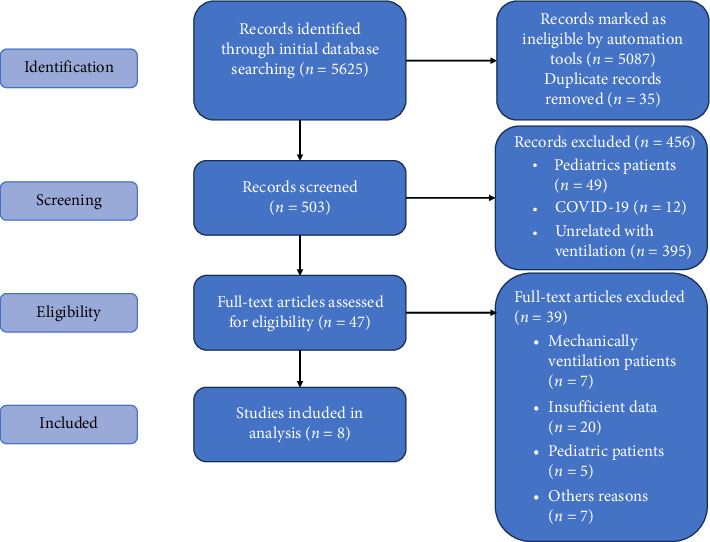
Research flowchart according to the PRISMA statement.

After reviewing abstracts, 47 papers were directly related to manual ventilation and its effectiveness. Of these, only eight provided clear, detailed, and accurate methods for evaluating ventilation effectiveness and analyzing the resulting data. The study protocol was presented on Figure 1.

Five of the eight included papers were designed as simulation studies [14–18], while three were clinical trials [19–21]. In laboratory-based studies, following parameters were collected: tidal volume (Vt), respiratory rate (Vr), peak lung pressure (Ppeak), inspiratory time (Itime), expiratory time (Etime), and gastric volume (Vg). In clinical trials, additional measurements were recorded, including blood oxygen saturation (SpO2), end-tidal carbon dioxide concentration (EtCO2), exhaled air volume (Vte), minute ventilation (VE), and peak inspiratory pressure (PIP). The recommended ventilation parameters, as outlined by ERC and AHA, were summarized in Table 1.

In most of the reviewed studies, Vt and Vr were used to assess the effectiveness of ventilation. Notably, there was significant variation in tolerance ranges adopted across studies. In five studies, a single breath was considered correct if its volume fell within a specific range, typically between 250 mL and 700 mL [14, 16–18, 21]. Of these, three studies also allowed an alternative measurement based on the recommended milliliters per kilogram of body weight, which ranged from 4 to 8 mL/kg [14, 17, 18]. In two studies, Vt was deemed correct if visible chest rise was observed, while one study defined correct inspiration as at least 4 mL/kg, assuming dead space ventilation of less than 150 mL [20].

Variability was also observed in the acceptable Vr In five studies, the range for appropriate respiratory rates was between 8 and 16 breaths per minute [14–18]. In addition to Vt, one study also assessed the EtCO2 level, considering a value greater than 3.5 kPa to be correct [21]. Another study evaluated minute ventilation along with Vt and Vr, considering it correct if it fell within the range of 5.28–6.96 L per minute [18].

In seven studies, participants ventilated the patient for a specified duration time (ranging from 60 to 480 s), completing the task only once [14–18, 20, 21]. In one study, participants performed the task twice, with two 90 s trials [19]. Detailed results were presented in Table 2.

None of the included studies presented the effect of ventilation efficiency on patient survival outcomes.

## 4. Discussion

The authors of this study aimed to analyze methods reported in the literature that are useful for assessing manual ventilation. Of the 8 studies included in this review, 5 were simulation based and 3 clinically based. All referenced AHA or ERC guidelines but selection of outcome measures differed. Other variables selected as outcome measures included Vr, Vt, Vg, Itime, and EtCO2. Only in two studies, Gruber et al. [19] and Zobrist, Casmaer, and April [15], chest rising was proposed as a determinant of adequate ventilation despite measured tidal volume.

The aims of studies varied, with 3 focused on the conduct of manual ventilation with a BVM among professional groups [14, 18, 19], 2 focused on evaluating products designed to guide proper ventilation [16, 17], 2 comparing the use of two products [15, 21], and 1 evaluating a new process [20]. According to definitions and methods used to assess ventilation efficiency, despite clear AHA and ERC guidelines, many researchers have adopted different tolerance ranges for defining adequate ventilation and have not consistently applied the same criteria during laboratory testing.

Notably, variations were observed in study methodologies regarding the length of time for which measures were recorded, with some studies reporting a duration as short as 1 min [20, 21]. In others, variables were summed across the measurement interval with ranged from 1 to 5 min [14–19].

Three studies reported hypoventilation and/or hyperventilation associated with the ventilation technique [14, 16, 17]. Outcomes related to survival were not reported in any of the studies, likely due to the multitude of factors that can influence this outcome in clinical practice. In the study by Gruber et al. [19], manual ventilation was reported to have failed in 34% of the cases based on the assumptions made by the authors. Accurate results were obtained in the research by Shaikh et al. [21]. In this case, the percentage of normal ventilations was determined to be 58%, assuming that 2/3 of the breaths are normal and EtCO2 > 3.5 kPa.

In the case of the study by Joffe et al. [20], the percentage of successful ventilations was not determined. However, it was noted that the administration of a muscle neuroblockade may increase tidal volume delivered during ventilation. Only in the simulation study by Zobrist, Casmaer, and April [15], clear quantifiable values for the percentage of normal ventilation were provided. This study aimed to support the authors in determining specific volumes delivered by study participants.

This literature review highlights a partial compromise between the AHA and the ERC regarding recommended ventilation parameters. While experts affiliating these organizations go along with respiratory rate of 10 breaths per minute, they disagree on the recommended tidal volume. The AHA suggests fixed tidal volume of 500–600 mL, regardless of the patient body weight, whereas the ERC bases tidal volume on body weight, recommending 6–8 mL per kilogram. Both organizations acknowledge the difficulty in establishing strict tolerance ranges for effective ventilation due to variations in patient physiology. As a result, they have accepted visible chest rise as a practical indicator of ventilation effectiveness [12, 13].

International guidelines recommend monitoring both average ventilation rate and average tidal volume, and it is a standard practice to assess both parameters simultaneously to evaluate normal ventilation [12, 13]. However, only about half of the reviewed studies used such a correlation. Moreover, various methods were utilized to analyze ventilation performance. The most commonly used approach was the overall mean value method, which calculates the average value of each variable by combining all tests, regardless of the sample size [15, 16, 18–21]. This method, however, may be inadequate, as it fails to account for interindividual and intraindividual variability in ventilation parameters. Ventilation performance can vary significantly both between test subjects and across different ventilation cycles.

The second method, used in two studies [14, 17], is a novel approach developed by Khoury et al. [14]. They identified the lack of a standardized method for evaluating the effectiveness of guided ventilation that accounts for inter- and intrapersonal variability in his systematic review [22]. In the result, Khoury et al. proposed another method assessing ventilation performance in one-minute window, based on Vt and Vr, with analysis occurring every three ventilation cycles. Each one-minute window may be classified into one of the three groups: (1) effective ventilation: Vr ≤ 15 per minute, Vt ≤ 600 mL, and/or at least 8 adequate ventilation cycles with Vt between 300 and 600 mL. (2) Inadequate ventilation: Vr ≤ 15 per minute, average Vt ≤ 600 mL, and/or fewer than 8 adequate ventilation cycles with Vt between 300 and 600 mL. (3) Excessive ventilation: Vr > 15 per minute and/or Vt > 600 mL. This method is considered highly objective as it accounts for both inter- and intrapersonal variability, providing a more precise evaluation of ventilation effectiveness.

Both manikin studies and clinical trials have their advantages and disadvantages. A study by De Luca et al. [23] demonstrated that the use of manikins can influence measurements of peak pressure, tidal volume, and leakage, depending on the experimental model. Therefore, it is essential to evaluate the manikin's internal resistance and airway dead space. Similarly, a study by Hesselfeldt, Kristensen, and Rasmussen [24] found the SimMan 3G human patient simulator (Laerdal, Stavanger, Norway) to be generally realistic though it may differ from human airways in critical ways.

However, clinical trials face limitations, including the need to recruit sufficient personnel and patients, and the inherent risks of adverse reactions, including serious health issues or death. In addition, it is more challenging to create a fully controlled environment in clinical settings, which can hinder the ability to obtain comparable data across studies. Manikin-based research, on the other hand, eliminates the need for ethics committee approval in some countries, as well as the necessity of patient recruitment. It also avoids the risks associated with human trials [25]. Manikins allow for stable, controlled, and repeatable conditions, which are advantageous for comparative research. However, even the most advanced high-fidelity manikins cannot fully replicate the tactile sensations or fine anatomical differences present in real patients.

The limitations of simulation studies compared with clinical trials were discussed by Rai and Popat [26], with a preference suggested for clinical studies. This preference arises because results obtained from manikin studies often require confirmation through subsequent clinical trials. However, the use of manikins is considered valuable in cases where specific study conditions cannot be safely or practically replicated in human patients.

### 4.1. Limitations

This review has several limitations. First, making direct comparisons between studies is challenging due to the diverse objectives set by the researchers, which leads to varied results. Second, studies published in languages other than English were excluded, potentially limiting the scope of the data presented. Third, there was significant variation in the variables used to assess ventilation effectiveness. Clearly defining evaluation criteria and corresponding analysis methods is crucial for accurate interpretation of study results. This is especially important in laboratory studies, where it is difficult to correlate ventilation data with physiological responses.

### 4.2. Implication for Further Studies

When planning, executing, and evaluating simulation studies, it is important to consider the reliability of the results, given the characteristics of manikins and their potential impact on ventilation measurements. Manikins allow for stable, controlled, and repeatable conditions, which are advantageous for comparative research. However, even the most advanced high-fidelity manikins cannot fully replicate the tactile sensations or fine anatomical differences present in real patients.

In clinical trials, researchers could correlate Vt and Vr with physiological data such as EtCO2 or SpO2, allowing for a more accurate assessment of ventilation effectiveness.

To evaluate the compliance of a product application or ventilation process with ERC and AHA guidelines, it is considered reasonable to assess at least Vt and Vr, regardless of the purpose of the study. Two methods of analysis were highlighted in the studies reviewed, with the method proposed by Khoury et al. appearing more precise and objective due to its consideration of intra- and interindividual variability.

## 5. Conclusions

Recent studies have defined the effectiveness of manual ventilation in different ways. ERC and AHA guidelines, which could serve as a foundation for these studies, remain inconclusive. This has led to multiple definitions of effective ventilation and inconsistency in the variables used in the research. Assessing the quality of ventilation by a single parameter may be not accurate. It is necessary to permanently merge ventilation rate and ventilation volume in order to properly assess ventilation. Dividing the entire study period into smaller ranges may give a more accurate view of the ventilation presented.

There is a need for studies involving humans to corelate ventilation rate and volume with physiological parameters. In addition, standardizing the methods used by researchers would facilitate better comparison of results across studies.

## Figures and Tables

**Table 1:** Comparison between selected ventilation parameters in ERC and AHA guidelines.

	Year of publication	Tidal volume	Respiratory rate
AHA	2015	6–7 mL/kg b.w.	8–10
ERC	2015	500–600 (6–7 mL/kg b.w.)	10
AHA	2020	500–600 mL or chest rise	10
ERC	2021	6–8 mL/kg m.c. or chest rise	10

Abbreviations: AHA, American Heart Association; ERC, European Resuscitation Council.

**Table 2:** Characteristics of studies included in the review.

Authors'	Publication year	Type of research	Number of participants	Method of analysis	Parameters analyzed	Parameters defining correct ventilation				
						Vt	Vr (min)	Vte	EtC02 (kPa)	Ve
Gruber et al. [19]	2014	Clinical	150	Overall mean value	Vt, Vg	Visible chest rise				
Joffe et al. [20]	2015	Clinical	210	Overall mean value	Vte, VE			> 4 mL/kg when Vds < 150 mL/breath		
Shaikh Robinson, and Hasan [21]	2016	Clinical	60	Overall mean value	Vt, EtCO2	> 250 mL in 2/3 of breaths			> 3.5	
Khoury et al. [14]	2016	Simulation	140	Authors'	Vt, Vr	300–600 mL, 4–8 mL/kg	8–15			
Zobrist Casmaer, and April [15]	2016	Simulation	70	Overall mean value	Vt	Visible chest rise	10			
Kim et al. [16]	2018	Simulation	121	Overall and individual mean value	Vr, Vt, Itime	400–700 mL	8–12			
Khoury et al. [17]	2019	Simulation	40	Authors'	Vt, Vr	300–600 mL, 4–8 mL/kg	8–15			
Culbreth and Gardenhire [18]	2020	Simulation	98	Overal mean value	Vt Ppeak, Itime, Irise time, flow rate	440–580 mL, 6–8 mL/kg	12			5.28–6.96 L/min

## Data Availability

The data of this study are available from the corresponding author upon reasonable request.

## References

[1] Aufderheide T. P., Lurie K. G. (2004). Death by Hyperventilation: A Common and Life-Threatening Problem During Cardiopulmonary Resuscitation. *Critical Care Medicine*.

[2] Komatsu R., Kasuya Y., Yogo H. (2010). Learning Curves for Bag-and-Mask Ventilation and Orotracheal Intubation. *Anesthesiology*.

[3] Dorph E., Wik L., Strømme T. A., Eriksen M., Steen P. A. (2004). Oxygen Delivery and Return of Spontaneous Circulation With Ventilation: Compression Ratio 2:30 Versus Chest Compressions Only CPR in Pigs. *Resuscitation*.

[4] Idris A. H., Banner M. J., Wenzel V., Fuerst R. S., Becker L. B., Melker R. J. (1994). Ventilation Caused by External Chest Compression Is Unable to Sustain Effective Gas Exchange During CPR: A Comparison With Mechanical Ventilation. *Resuscitation*.

[5] Kill C., Torossian A., Freisburger C. (2009). Basic Life Support With Four Different Compression/Ventilation Ratios in a Pig Model: The Need for Ventilation. *Resuscitation*.

[6] Boussarsar M., Thierry G., Jaber S., Roudot-Thoraval F., Lemaire F., Brochard L. (2002). Relationship Between Ventilatory Settings and Barotrauma in the Acute Respiratory Distress Syndrome. *Intensive Care Medicine*.

[7] Stone B. J., Chantler P. J., Baskett P. J. F. (1998). The Incidence of Regurgitation during Cardiopulmonary Resuscitation: A Comparison Between the Bag Valve Mask and Laryngeal Mask Airway. *Resuscitation*.

[8] Weiler N., Heinrichs W., Dick W. (1995). Assessment of Pulmonary Mechanics and Gastric Inflation Pressure During Mask Ventilation. *Prehospital and Disaster Medicine*.

[9] Wenzel V., Idris A. H., Banner M. J., Kubilis P. S., Williams J. L. (1998). Influence of Tidal Volume on the Distribution of Gas Between the Lungs and Stomach in the Nonintubated Patient Receiving Positive-Pressure Ventilation. *Critical Care Medicine*.

[10] Link M. S., Berkow L. C., Kudenchuk P. J. (2015). Part 7: Adult Advanced Cardiovascular Life Support: 2015 American Heart Association Guidelines Update for Cardiopulmonary Resuscitation and Emergency Cardiovascular Care. *Circulation*.

[11] Monsieurs K. G., Nolan J. P., Bossaert L. L. (2015). European Resuscitation Council Guidelines for Resuscitation 2015. *Resuscitation*.

[12] Panchal A. R., Bartos J. A., Cabañas J. G. (2020). Part 3: Adult Basic and Advanced Life Support: 2020 American Heart Association Guidelines for Cardiopulmonary Resuscitation and Emergency Cardiovascular Care. *Circulation*.

[13] Perkins G. D., Gräsner J. T., Semeraro F. (2021). European Resuscitation Council Guidelines 2021: Executive Summary. *Resuscitation*.

[14] Khoury A., Sall F. S., De Luca A. (2016). Evaluation of Bag-Valve-Mask Ventilation in Manikin Studies: What Are the Current Limitations?. *BioMed Research International*.

[15] Zobrist B., Casmaer M., April M. D. (2016). Single Rescuer Ventilation Using a Bag-Valve Mask With Internal Handle: A Randomized Crossover Trial. *The American Journal of Emergency Medicine*.

[16] Kim J. H., Beom J. H., You J. S., Cho J., Min I. K., Chung H. S., Szyld E. (2018). Effect of Flashlight Guidance on Manual Ventilation Performance in Cardiopulmonary Resuscitation: A Randomized Controlled Simulation Study. *PLoS One*.

[17] Khoury A., De Luca A., Sall F. S., Pazart L., Capellier G. (2019). Ventilation Feedback Device for Manual Ventilation in Simulated Respiratory Arrest: A Crossover Manikin Study. *Scandinavian Journal of Trauma, Resuscitation and Emergency Medicine*.

[18] Culbreth R. E., Gardenhire D. S. (2021). Manual Bag Valve Mask Ventilation Performance Among Respiratory Therapists. *Heart and Lung*.

[19] Gruber E., Oberhammer R., Balkenhol K. (2014). Basic Life Support Trained Nurses Ventilate More Efficiently With Laryngeal Mask Supreme Than With Facemask or Laryngeal Tube Suction-Disposable—A Prospective, Randomized Clinical Trial. *Resuscitation*.

[20] Joffe A. M., Ramaiah R., Donahue E. (2015). Ventilation by Mask Before and After the Administration of Neuromuscular Blockade: A Pragmatic Non-Inferiority Trial. *BMC Anesthesiology*.

[21] Shaikh A., Robinson P. N., Hasan M. (2016). The Tulip GT Airway Versus the Facemask and Guedel Airway: A Randomised, Controlled, Cross-Over Study by Basic Life Support-Trained Airway Providers in Anaesthetised Patients. *Anaesthesia*.

[22] Khoury A., De Luca A., Sall F. S., Pazart L., Capellier G. (2015). Performance of Manual Ventilation: How to Define Its Efficiency in Bench Studies? A Review of the Literature. *Anaesthesia*.

[23] De Luca A., Sall F. S., Sailley R., Capellier G., Khoury A. (2015). Reliability of Manikin‐Based Studies: An Evaluation of Manikin Characteristics and Their Impact on Measurements of Ventilatory Variables. *Anaesthesia*.

[24] Hesselfeldt R., Kristensen M. S., Rasmussen L. S. (2005). Evaluation of the Airway of the SimMan Full‐Scale Patient Simulator. *Acta Anaesthesiologica Scandinavica*.

[25] Cann C., Hall J. E., Sudheer P. S., Turley A. (2005). Is Ethical Approval Necessary for Manikin Studies?. *Anaesthesia*.

[26] Rai M. R., Popat M. T. (2011). Evaluation of Airway Equipment: Man or Manikin?. *Anaesthesia*.

